# Supplementary material for: Orbital modulation of subtropical versus subantarctic moisture sources in the southeast Pacific mid-latitudes
Source: Nat Commun. 2024 Aug 29;15:7512. doi: 10.1038/s41467-024-51985-4 (PMC11362560; doi:10.1038/s41467-024-51985-4)
Supplement: Supplementary file 1 — Supplemetary Information [file 41467_2024_51985_MOESM1_ESM.pdf]

Supplementary Materials for

Orbital modulation of subtropical versus subantarctic moisture sources in the southeast Pacific mid-latitudes

Jérôme Kaiser[a*], Enno Schefuß[b], James Collins[c#], René Garreaud[d,e], Jan-Berend W. Stuut[b,f], Nicoletta Ruggieri[c], Ricardo De Pol-Holz[g] and Frank Lamy[c]

[a]Leibniz Institute for Baltic Sea Research (IOW), 18119 Rostock-Warnemünde, Germany; jerome.kaiser@io-warnemuende.de

[b]MARUM – Center for Marine Environmental Sciences, Bremen University, 28359 Bremen, Germany

[c]Alfred-Wegener-Institut Helmholtz-Zentrum für Polar- und Meeresforschung (AWI), 27570 Bremerhaven, Germany

[d]Center for Climate and Resilience Research (CR)[2], University of Chile, Santiago, Chile

[e]Department of Geophysics, University of Chile, Santiago, Chile

[f]NIOZ – Royal Netherlands Institute for Sea Research, Department of Ocean Systems, and VU - Vrije Universiteit Amsterdam, Department of Earth Sciences, Amsterdam, the Netherlands

[g]Centro de Investigación GAIA-Antártica (CIGA), University of Magallanes, Magallanes, Chile

[#]*present address: Thermo Fisher Scientific (Bremen) GmbH, Hanna-Kunath-Str. 11, 28199 Bremen, Germany*

*Corresponding author: jerome.kaiser@io-warnemuende.de

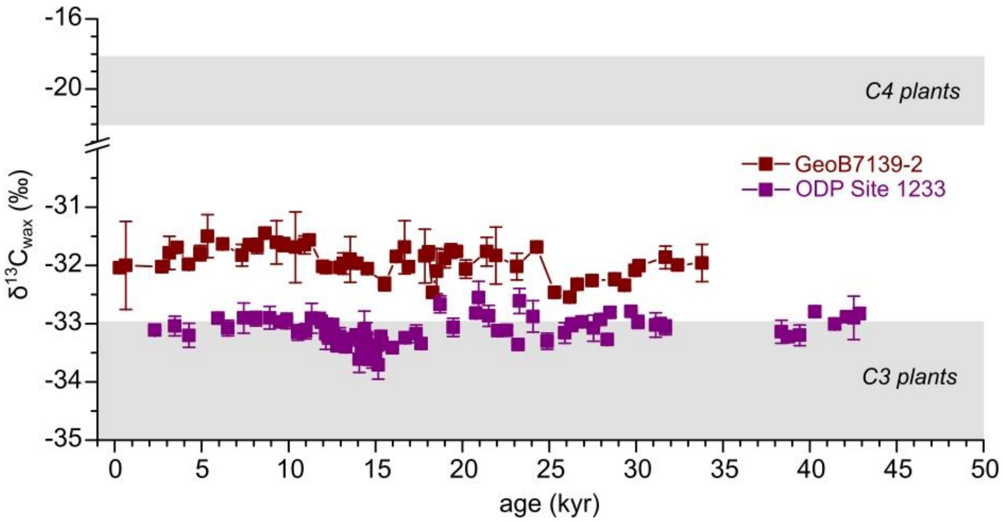

Figure S1. **Carbon isotope record of the *n*-C_{31} alkane ($\delta^{13}C_{wax}$) in core GeoB7139-2 and ODP Site 1233**. The horizontal grey bars represent the range of *n*-alkanes $\delta^{13}C$ values of C_3 and C_4 plants[1]. Note the break in the ordinate.

Table S1. **Weighted mean of deuterium excess and deuterium in meteoric water at La Serena (30°S) and Puerto Montt (41°S) meteorological stations**. Data from the Global Network of Isotopes in Precipitation (GNIP; www.iaea.org/water).

Site	lat N	long E	altitude (m)	first year of observation	last year of observation	weighted mean deuterium excess (‰)	weighted mean deuterium (‰)	Rain amount (mm)
La Serena	-30	-71	146	1988	2007	13.9	-34	89
Puerto Montt (La Chamiza)	-41	-73	13	1964	2007	9.8	-44	1670

Table S2. **$\delta^{13}C_{wax}$ dataset of core ODP1233.** Note that ODP Site 1233 age model was recalibrated (see Table S11).

Core	Age (cal ka BP)	Depth (m)	δ^{13}Cwax (‰)	error bar (‰)
ODP1233	2.298	3.7	-33.1	0.0
ODP1233	3.445	4.8	-33.0	0.2
ODP1233	4.269	5.6	-33.2	0.2
ODP1233	5.948	7.7	-32.9	0.0
ODP1233	6.515	8.0	-33.1	0.1
ODP1233	7.413	9.1	-32.9	0.3
ODP1233	8.126	9.9	-32.9	0.1
ODP1233	8.914	11.0	-32.9	0.2
ODP1233	9.509	12.1	-33.0	0.0

ODP1233	9.899	12.9	-33.0	0.1
ODP1233	10.535	13.9	-33.2	0.1
ODP1233	10.986	14.7	-33.1	0.1
ODP1233	11.329	15.5	-32.9	0.3
ODP1233	11.787	16.4	-32.9	0.1
ODP1233	11.902	16.7	-33.0	0.1
ODP1233	12.144	17.3	-33.2	0.3
ODP1233	12.289	17.7	-33.2	0.1
ODP1233	12.434	18.2	-33.0	0.0
ODP1233	12.543	18.6	-33.0	0.1
ODP1233	12.796	19.4	-33.4	0.1
ODP1233	12.941	19.9	-33.2	0.2
ODP1233	13.287	20.5	-33.4	0.1
ODP1233	13.493	20.8	-33.3	0.0
ODP1233	13.906	21.2	-33.2	0.1
ODP1233	14.073	21.5	-33.6	0.2
ODP1233	14.212	21.8	-33.5	0.1
ODP1233	14.279	22.0	-33.4	0.2
ODP1233	14.369	22.3	-33.1	0.3
ODP1233	14.482	22.6	-33.2	0.3
ODP1233	14.571	22.8	-33.4	0.2
ODP1233	14.676	23.1	-33.6	0.1
ODP1233	14.830	23.5	-33.6	0.2
ODP1233	15.009	24.0	-33.5	0.1
ODP1233	15.167	24.4	-33.7	0.2
ODP1233	15.324	24.8	-33.2	0.0
ODP1233	15.369	24.9	-33.3	0.1
ODP1233	15.973	25.8	-33.4	0.1
ODP1233	16.715	26.7	-33.2	0.0
ODP1233	17.344	27.5	-33.2	0.2
ODP1233	17.634	27.8	-33.3	0.1
ODP1233	18.715	29.2	-32.7	0.1
ODP1233	19.489	30.3	-33.1	0.2
ODP1233	20.792	33.1	-32.8	0.1
ODP1233	20.938	33.5	-32.6	0.3
ODP1233	21.516	34.7	-32.9	0.2
ODP1233	22.051	35.8	-33.1	0.0
ODP1233	22.560	36.8	-33.1	0.1
ODP1233	23.211	38.2	-33.4	0.0
ODP1233	23.297	38.3	-32.6	0.2
ODP1233	24.082	40.3	-32.9	0.3
ODP1233	24.891	42.1	-33.3	0.1
ODP1233	25.899	43.8	-33.2	0.2
ODP1233	26.311	44.6	-33.0	0.1

ODP1233	26.896	45.6	-33.0	0.0
ODP1233	27.558	46.5	-33.1	0.2
ODP1233	27.961	47.0	-32.9	0.1
ODP1233	28.350	47.6	-33.3	0.0
ODP1233	28.516	47.9	-32.8	0.1
ODP1233	29.712	51.2	-32.8	0.0
ODP1233	30.114	51.8	-33.0	0.1
ODP1233	31.123	53.5	-33.0	0.2
ODP1233	31.411	54.0	-33.0	0.0
ODP1233	31.700	54.5	-33.1	0.1
ODP1233	38.363	63.3	-33.1	0.2
ODP1233	38.780	64.0	-33.2	0.0
ODP1233	39.421	65.1	-33.2	0.2
ODP1233	40.302	66.4	-32.8	0.1
ODP1233	41.443	68.4	-33.0	0.1
ODP1233	42.123	70.3	-32.9	0.0
ODP1233	42.537	71.5	-32.9	0.4
ODP1233	42.857	72.5	-32.8	0.1

Table S3. **δD_{wax} dataset of core ODP1233/GeoB7196-1.** Note that ODP Site 1233 age model was recalibrated (see Table S11).

Core	Age (cal ka BP)	Depth (m)	δDwax (‰)	error bar (‰)
GeoB7196-1	0.407	0.4	-165.8	0.7
GeoB7196-1	0.898	0.8	-163.8	0.6
GeoB7196-1	1.218	1.2	-164.1	1.3
GeoB7196-1	1.527	1.6	-163.7	0.4
GeoB7196-1	1.573	1.6	-163.6	0.3
GeoB7196-1	1.620	1.7	-163.7	0.5
GeoB7196-1	1.713	1.8	-163.9	0.5
GeoB7196-1	1.771	1.8	-163.4	1.0
ODP1233	2.298	3.7	-164.1	0.6
GeoB7196-1	2.735	2.6	-162.5	0.9
ODP1233	3.445	4.8	-161.2	0.0
GeoB7196-1	3.914	3.6	-160.9	0.7
ODP1233	4.269	5.6	-165.1	0.1
GeoB7196-1	4.926	4.6	-158.0	2.0
GeoB7196-1	5.709	5.6	-160.4	1.1
ODP1233	6.515	8.0	-166.5	0.5
GeoB7196-1	6.587	6.6	-161.3	0.0
ODP1233	7.413	9.1	-166.6	1.0
ODP1233	8.126	9.9	-168.5	1.3
ODP1233	8.914	11.0	-166.2	0.5

ODP1233	9.509	12.1	-166.0	0.2
ODP1233	9.899	12.9	-162.3	2.4
ODP1233	10.535	13.9	-166.1	1.0
ODP1233	10.986	14.7	-159.0	1.4
ODP1233	11.329	15.5	-166.1	0.5
ODP1233	11.787	16.4	-164.5	2.1
ODP1233	11.902	16.1	-167.8	0.1
ODP1233	12.144	17.3	-165.0	3.7
ODP1233	12.289	17.7	-166.0	0.3
ODP1233	12.434	18.2	-166.0	0.5
ODP1233	12.543	18.6	-167.4	1.4
ODP1233	12.796	19.4	-167.3	0.5
ODP1233	13.287	20.5	-166.9	0.0
ODP1233	13.493	20.8	-166.2	1.0
ODP1233	13.906	21.2	-166.1	1.5
ODP1233	14.073	21.5	-169.0	0.9
ODP1233	14.212	21.8	-169.7	1.0
ODP1233	14.279	22.0	-167.8	1.4
ODP1233	14.369	22.3	-170.1	2.8
ODP1233	14.482	22.6	-168.1	2.3
ODP1233	14.571	22.8	-169.7	0.5
ODP1233	14.676	23.1	-169.8	0.1
ODP1233	14.830	23.5	-169.3	0.2
ODP1233	15.009	24.0	-170.4	0.3
ODP1233	15.167	24.4	-169.2	0.6
ODP1233	15.324	24.8	-164.3	1.4
ODP1233	15.369	24.9	-172.9	0.2
ODP1233	15.973	25.8	-172.5	0.1
ODP1233	16.715	26.7	-169.8	0.3
ODP1233	17.344	27.5	-173.3	0.9
ODP1233	18.715	29.2	-170.8	2.4
ODP1233	19.489	30.3	-175.6	1.1
ODP1233	20.792	33.1	-173.9	4.0
ODP1233	21.516	34.7	-172.9	0.8
ODP1233	22.051	35.8	-174.0	0.8
ODP1233	22.560	36.8	-173.5	0.2
ODP1233	23.211	38.2	-171.7	0.6
ODP1233	24.082	40.3	-170.8	0.4
ODP1233	24.456	41.4	-180.1	0.5
ODP1233	24.891	42.1	-173.2	1.5
ODP1233	25.344	42.8	-177.8	0.1
ODP1233	25.899	43.8	-176.8	0.9
ODP1233	26.311	44.6	-183.6	0.6
ODP1233	26.896	45.6	-182.0	0.6

ODP1233	27.558	46.5	-175.0	0.7
ODP1233	27.961	47.0	-181.9	2.1
ODP1233	28.350	47.6	-178.5	0.0
ODP1233	28.516	47.9	-179.0	1.7
ODP1233	29.712	51.2	-176.2	0.4
ODP1233	30.114	51.8	-182.0	0.3
ODP1233	31.123	53.5	-182.8	0.7
ODP1233	31.411	54.0	-189.2	1.1
ODP1233	31.700	54.5	-188.0	0.7
ODP1233	33.216	56.4	-180.6	0.0
ODP1233	36.766	60.9	-176.2	0.0
ODP1233	37.288	61.6	-177.0	2.9
ODP1233	37.847	62.4	-183.2	0.0
ODP1233	38.363	63.3	-176.8	0.5
ODP1233	38.780	64.0	-176.7	0.6
ODP1233	39.421	65.1	-179.0	0.4
ODP1233	40.302	66.4	-180.1	0.2
ODP1233	41.443	68.4	-179.5	0.0
ODP1233	42.123	70.3	-178.9	0.3
ODP1233	42.537	71.5	-174.6	1.1
ODP1233	42.857	72.5	-179.1	0.7
ODP1233	43.534	74.3	-173.5	0.0
ODP1233	44.128	75.7	-173.6	0.0

Table S4. **$\delta^{13}C_{wax}$ dataset of core GeoB7139-2.** Note that the age model was recalibrated (see Table S12).

Age (cal ka BP)	Depth (cm)	δ^{13}Cwax (‰)	error bar (‰)
0.290	2	-32.0	0.1
0.630	4	-32.0	0.8
2.710	18	-32.0	0.1
3.149	22	-31.8	0.3
3.588	26	-31.7	0.1
4.247	32	-32.0	0.0
4.906	38	-31.8	0.1
5.345	42	-31.5	0.4
6.224	50	-31.6	0.0
7.322	60	-31.8	0.2
7.762	64	-31.6	0.0
8.201	68	-31.7	0.1
8.640	72	-31.4	0.0
9.299	78	-31.6	0.4
9.739	82	-31.6	0.1

10.398	88	-31.7	0.6
10.923	96	-31.6	0.2
11.186	100	-31.6	0.1
11.975	112	-32.0	0.0
12.172	115	-32.0	0.1
12.961	127	-32.0	0.0
13.158	130	-32.0	0.2
13.552	136	-31.9	0.4
13.947	142	-32.0	0.1
14.538	151	-32.1	0.0
15.531	168	-32.3	0.1
16.223	180	-31.9	0.1
16.685	188	-31.7	0.5
16.915	192	-32.0	0.0
17.838	208	-31.8	0.5
18.069	212	-31.8	0.1
18.300	216	-32.5	0.1
18.530	220	-32.1	0.4
18.992	228	-31.9	0.2
19.337	234	-31.8	0.1
19.682	240	-31.8	0.1
20.200	249	-32.1	0.2
21.408	270	-31.8	0.2
21.926	279	-31.8	0.5
23.134	300	-32.0	0.2
24.285	320	-31.7	0.1
25.321	340	-32.5	0.1
26.185	360	-32.5	0.1
26.617	370	-32.3	0.0
27.480	390	-32.3	0.1
28.776	420	-32.2	0.1
29.353	430	-32.3	0.1
29.937	440	-32.1	0.0
30.170	444	-32.0	0.1
31.689	470	-31.9	0.2
32.389	482	-32.0	0.0
33.791	506	-32.0	0.3

Table S5. **δD_{wax} dataset of core GeoB7139-2.** Note that the age model was recalibrated (see Table S12).

Age (cal ka BP)	Depth (cm)	δDwax (‰)	error bar (‰)
0.120	1	-171.3	0.0
0.290	2	-167.3	0.2
0.630	4	-167.6	1.3

0.970	6	-164.5	1.6
2.270	14	-163.1	0.3
2.490	16	-161.0	1.0
2.710	18	-162.2	1.3
3.149	22	-161.1	0.4
3.588	26	-160.0	0.5
4.028	30	-162.4	0.7
4.467	34	-158.6	0.7
4.906	38	-158.6	0.6
5.345	42	-160.1	0.0
5.675	45	-158.2	2.5
5.785	46	-157.3	0.0
6.004	48	-158.7	1.0
6.224	50	-156.0	0.9
6.554	53	-155.0	2.6
7.103	58	-159.3	1.4
7.322	60	-155.8	1.3
7.762	64	-156.9	1.5
7.981	66	-157.5	0.2
8.201	68	-154.2	1.0
8.640	72	-154.9	0.4
9.080	76	-158.6	1.2
9.299	78	-155.7	1.1
9.739	82	-152.6	0.8
10.178	86	-154.7	0.7
10.398	88	-152.6	0.5
10.660	92	-153.0	0.0
10.923	96	-153.5	1.1
11.055	98	-154.7	0.6
11.186	100	-152.1	0.6
11.383	103	-159.2	0.0
11.778	109	-154.8	0.0
11.975	112	-155.4	0.9
12.172	115	-155.8	0.8
12.567	121	-156.7	0.0
12.961	127	-156.3	0.1
13.158	130	-154.3	1.1
13.355	133	-156.8	1.1
13.552	136	-153.8	0.3
13.750	139	-155.0	0.1
13.947	142	-155.1	0.8
14.341	148	-158.7	0.3
14.538	151	-160.5	0.2
14.724	154	-159.4	1.0

14.897	157	-158.9	0.7
15.300	164	-159.1	1.4
15.531	168	-160.8	1.0
15.992	176	-162.4	0.7
16.223	180	-162.6	0.1
16.454	184	-168.8	2.5
16.685	188	-160.1	0.7
16.915	192	-158.2	0.1
17.146	196	-162.6	0.9
17.261	198	-164.7	0.2
17.377	200	-163.1	0.8
17.607	204	-163.6	0.0
17.838	208	-162.3	0.7
18.069	212	-162.5	0.3
18.300	216	-165.2	2.5
18.530	220	-165.5	0.1
18.761	224	-169.5	0.4
18.992	228	-165.2	1.2
19.222	232	-167.1	0.2
19.337	234	-167.4	0.4
19.452	236	-165.0	0.1
19.682	240	-165.0	1.8
20.200	249	-166.7	0.1
20.545	255	-166.4	0.2
20.833	260	-161.3	0.0
21.408	270	-169.9	0.4
21.696	275	-169.3	1.2
21.926	279	-164.6	0.5
22.559	290	-167.0	0.3
23.134	300	-169.2	1.3
23.652	309	-162.1	0.1
24.285	320	-167.5	0.6
24.860	330	-167.7	0.9
25.321	340	-167.3	0.3
25.753	350	-170.8	0.7
26.185	360	-169.3	0.8
26.617	370	-170.8	1.1
27.048	380	-175.3	1.2
27.480	390	-167.0	3.7
27.912	400	-174.6	2.7
28.344	410	-172.1	0.6
28.776	420	-165.8	0.8
29.353	430	-168.2	1.8
29.703	436	-160.5	0.7

29.937	440	-164.3	0.9
30.170	444	-163.1	0.4
31.105	460	-164.2	0.1
31.689	470	-162.7	0.2
32.389	482	-163.8	0.9
33.090	494	-162.1	0.6
33.791	506	-164.8	0.5
34.748	518	-162.5	0.2
36.216	530	-158.8	0.6
37.685	542	-163.2	0.8
38.664	550	-162.2	1.0
39.154	554	-161.6	0.7
39.888	560	-162.3	0.5
40.010	561	-161.1	0.8
40.500	565	-163.0	0.3
40.745	567	-161.7	0.7
41.357	572	-164.4	0.7
41.846	576	-163.8	0.5
42.213	579	-166.6	0.6
42.703	583	-166.7	1.6
43.315	588	-167.3	0.1
43.804	592	-167.4	0.6
44.294	596	-166.6	0.8
44.416	597	-165.9	0.2
44.783	600	-163.9	0.2
45.151	603	-167.1	0.5
45.640	607	-164.8	1.7
46.007	610	-167.0	0.5
46.619	615	-169.2	0.9
47.231	620	-169.7	0.3
47.843	625	-166.7	0.6
48.455	630	-169.1	0.6

Table S6. **$U^{K'}_{37}$ SST dataset of core ODP1233.** The data were published in a previous study[2]. Note that ODP Site 1233 age model was recalibrated (see Table S11).

Core	Age (cal ka BP)	Depth (m)	UK'37 SST (°C)
ODP1233	-0.050	0.0	13.5
ODP1233	0.190	0.7	13.2
ODP1233	0.431	1.4	13.8
ODP1233	0.765	2.2	13.8
ODP1233	1.388	2.9	13.8
ODP1233	2.231	3.6	13.6

ODP1233	3.017	4.3	13.8
ODP1233	3.736	5.1	13.7
ODP1233	4.382	5.8	13.8
ODP1233	4.674	6.5	13.8
ODP1233	5.096	7.2	14.5
ODP1233	5.339	7.4	14.5
ODP1233	6.473	8.0	14.4
ODP1233	7.077	8.7	14.0
ODP1233	7.773	9.5	14.0
ODP1233	8.378	10.2	14.3
ODP1233	8.865	10.9	14.5
ODP1233	9.282	11.7	14.9
ODP1233	9.672	12.4	14.8
ODP1233	9.931	12.9	15.4
ODP1233	10.077	13.2	14.9
ODP1233	10.275	13.5	15.4
ODP1233	10.350	13.6	15.4
ODP1233	10.498	13.8	14.4
ODP1233	10.721	14.2	15.5
ODP1233	10.895	14.6	15.2
ODP1233	11.014	14.8	15.1
ODP1233	11.129	15.0	15.2
ODP1233	11.186	15.2	15.0
ODP1233	11.243	15.3	14.4
ODP1233	11.301	15.4	15.0
ODP1233	11.358	15.5	14.9
ODP1233	11.415	15.6	14.9
ODP1233	11.472	15.8	14.9
ODP1233	11.530	15.9	14.4
ODP1233	11.587	16.0	14.5
ODP1233	11.644	16.1	14.6
ODP1233	11.701	16.2	14.7
ODP1233	11.758	16.4	14.5
ODP1233	11.816	16.5	14.3
ODP1233	11.873	16.6	14.3
ODP1233	11.930	16.7	15.0
ODP1233	11.987	16.8	14.3
ODP1233	12.045	17.0	14.1
ODP1233	12.090	17.1	13.8
ODP1233	12.198	17.4	13.6
ODP1233	12.271	17.7	13.8
ODP1233	12.307	17.8	13.4
ODP1233	12.343	17.9	12.7
ODP1233	12.380	18.0	13.0

ODP1233	12.416	18.2	12.8
ODP1233	12.452	18.3	12.8
ODP1233	12.488	18.4	12.9
ODP1233	12.525	18.5	12.9
ODP1233	12.561	18.6	12.7
ODP1233	12.597	18.8	12.9
ODP1233	12.633	18.9	12.9
ODP1233	12.670	19.0	13.4
ODP1233	12.706	19.1	12.5
ODP1233	12.742	19.2	13.1
ODP1233	12.815	19.5	12.8
ODP1233	12.851	19.6	12.7
ODP1233	12.887	19.7	12.7
ODP1233	12.923	19.8	12.7
ODP1233	12.959	20.0	12.8
ODP1233	12.996	20.1	12.2
ODP1233	13.032	20.2	12.6
ODP1233	13.124	20.3	12.9
ODP1233	13.227	20.4	13.1
ODP1233	13.330	20.6	13.1
ODP1233	13.442	20.7	12.4
ODP1233	13.545	20.8	12.8
ODP1233	13.648	20.9	12.6
ODP1233	13.751	21.1	12.2
ODP1233	13.854	21.2	12.7
ODP1233	14.051	21.4	12.8
ODP1233	14.096	21.5	13.2
ODP1233	14.141	21.7	13.0
ODP1233	14.167	21.7	12.8
ODP1233	14.212	21.8	13.0
ODP1233	14.257	22.0	12.9
ODP1233	14.302	22.1	12.8
ODP1233	14.347	22.2	12.6
ODP1233	14.392	22.3	12.7
ODP1233	14.437	22.4	12.8
ODP1233	14.482	22.6	12.3
ODP1233	14.526	22.7	12.7
ODP1233	14.571	22.8	13.1
ODP1233	14.616	22.9	12.7
ODP1233	14.661	23.0	12.9
ODP1233	14.717	23.2	12.3
ODP1233	14.762	23.3	12.1
ODP1233	14.807	23.4	12.6
ODP1233	14.852	23.6	12.7

ODP1233	14.897	23.7	12.5
ODP1233	14.942	23.8	12.6
ODP1233	14.987	23.9	12.2
ODP1233	15.032	24.0	12.3
ODP1233	15.077	24.2	12.9
ODP1233	15.122	24.3	12.2
ODP1233	15.167	24.4	13.2
ODP1233	15.212	24.5	12.6
ODP1233	15.257	24.6	12.1
ODP1233	15.301	24.8	11.6
ODP1233	15.346	24.9	11.7
ODP1233	15.391	25.0	11.8
ODP1233	15.441	25.1	12.1
ODP1233	15.489	25.2	12.2
ODP1233	15.586	25.3	12.6
ODP1233	15.682	25.4	12.0
ODP1233	15.731	25.5	11.8
ODP1233	15.779	25.5	12.3
ODP1233	15.876	25.7	12.4
ODP1233	15.973	25.8	11.8
ODP1233	16.086	25.9	10.9
ODP1233	16.182	26.0	10.9
ODP1233	16.279	26.2	10.6
ODP1233	16.376	26.3	11.1
ODP1233	16.473	26.4	10.8
ODP1233	16.570	26.5	10.6
ODP1233	16.666	26.6	10.0
ODP1233	16.763	26.8	10.4
ODP1233	16.860	26.9	9.5
ODP1233	16.957	27.0	9.8
ODP1233	17.053	27.1	10.5
ODP1233	17.150	27.2	10.1
ODP1233	17.247	27.4	10.3
ODP1233	17.344	27.5	9.7
ODP1233	17.441	27.6	9.3
ODP1233	17.537	27.7	9.2
ODP1233	17.634	27.8	8.4
ODP1233	17.731	28.0	8.4
ODP1233	17.826	28.1	7.6
ODP1233	17.912	28.2	8.5
ODP1233	18.007	28.3	8.4
ODP1233	18.101	28.4	8.8
ODP1233	18.196	28.5	9.1
ODP1233	18.290	28.7	8.5

ODP1233	18.384	28.8	8.8
ODP1233	18.479	28.9	8.7
ODP1233	18.573	29.0	8.3
ODP1233	18.857	29.4	9.1
ODP1233	19.223	29.9	8.9
ODP1233	19.422	30.2	10.5
ODP1233	19.555	30.5	9.0
ODP1233	19.755	30.8	9.7
ODP1233	19.971	31.2	9.2
ODP1233	20.157	31.6	9.9
ODP1233	20.306	31.9	9.3
ODP1233	20.464	32.3	9.5
ODP1233	20.618	32.7	9.3
ODP1233	20.767	33.0	9.7
ODP1233	20.867	33.3	9.5
ODP1233	20.917	33.4	8.9
ODP1233	21.026	33.6	9.8
ODP1233	21.201	34.0	8.7
ODP1233	21.259	34.1	8.8
ODP1233	21.434	34.5	9.9
ODP1233	21.609	34.8	8.4
ODP1233	21.784	35.2	9.5
ODP1233	21.900	35.4	7.1
ODP1233	21.959	35.6	7.3
ODP1233	22.134	35.9	8.5
ODP1233	22.308	36.3	9.0
ODP1233	22.483	36.6	9.2
ODP1233	22.594	36.9	9.4
ODP1233	22.767	37.2	8.5
ODP1233	22.907	37.5	8.1
ODP1233	23.027	37.8	8.3
ODP1233	23.143	38.0	9.0
ODP1233	23.259	38.3	9.7
ODP1233	23.374	38.5	10.1
ODP1233	23.432	38.6	9.9
ODP1233	23.548	38.9	10.2
ODP1233	23.663	39.1	10.2
ODP1233	23.726	39.2	10.4
ODP1233	23.842	39.5	10.6
ODP1233	23.916	39.7	9.2
ODP1233	23.949	39.8	10.3
ODP1233	23.982	39.9	9.5
ODP1233	24.082	40.3	10.7
ODP1233	24.148	40.5	10.1

ODP1233	24.184	40.7	8.8
ODP1233	24.283	41.0	8.0
ODP1233	24.383	41.3	8.7
ODP1233	24.456	41.4	8.7
ODP1233	24.677	41.8	9.1
ODP1233	24.824	42.0	9.6
ODP1233	24.897	42.1	9.5
ODP1233	25.344	42.8	9.5
ODP1233	25.804	43.6	8.3
ODP1233	26.126	44.2	8.3
ODP1233	26.187	44.3	8.2
ODP1233	26.249	44.4	8.1
ODP1233	26.496	44.9	9.5
ODP1233	26.558	45.0	8.9
ODP1233	26.993	45.8	9.3
ODP1233	27.574	46.5	10.7
ODP1233	28.138	47.2	10.2
ODP1233	28.516	47.9	10.0
ODP1233	28.575	48.0	9.6
ODP1233	28.634	48.1	9.9
ODP1233	28.707	48.3	9.8
ODP1233	28.766	48.4	9.9
ODP1233	28.825	48.5	10.0
ODP1233	28.869	48.6	9.8
ODP1233	28.883	48.6	10.5
ODP1233	28.942	48.8	10.9
ODP1233	28.996	48.9	10.3
ODP1233	29.055	49.0	10.2
ODP1233	29.114	49.1	10.2
ODP1233	29.139	49.2	10.4
ODP1233	29.164	49.4	10.3
ODP1233	29.215	49.6	9.9
ODP1233	29.240	49.7	9.9
ODP1233	29.266	49.8	9.6
ODP1233	29.291	50.0	9.0
ODP1233	29.316	50.1	10.2
ODP1233	29.342	50.2	10.2
ODP1233	29.367	50.3	10.1
ODP1233	29.392	50.4	10.2
ODP1233	29.418	50.6	9.7
ODP1233	29.443	50.7	9.8
ODP1233	29.496	50.8	10.3
ODP1233	29.568	50.9	9.6
ODP1233	29.640	51.0	10.2

ODP1233	29.712	51.2	10.1
ODP1233	29.784	51.3	10.0
ODP1233	29.856	51.4	9.7
ODP1233	29.928	51.5	9.6
ODP1233	30.000	51.6	10.8
ODP1233	30.072	51.8	10.3
ODP1233	30.114	51.8	10.4
ODP1233	30.150	51.9	9.9
ODP1233	30.186	51.9	10.0
ODP1233	30.222	52.0	10.1
ODP1233	30.264	52.1	9.7
ODP1233	30.336	52.2	9.8
ODP1233	30.408	52.3	10.0
ODP1233	30.480	52.4	9.8
ODP1233	30.553	52.6	9.2
ODP1233	30.625	52.7	8.9
ODP1233	30.697	52.8	9.0
ODP1233	30.769	52.9	8.3
ODP1233	30.841	53.0	10.1
ODP1233	30.913	53.2	9.3
ODP1233	30.985	53.3	8.2
ODP1233	31.057	53.4	8.1
ODP1233	31.123	53.5	7.9
ODP1233	31.195	53.6	7.7
ODP1233	31.267	53.7	8.9
ODP1233	31.339	53.9	8.8
ODP1233	31.411	54.0	9.6
ODP1233	31.483	54.1	9.2
ODP1233	31.556	54.2	9.4
ODP1233	31.628	54.3	9.1
ODP1233	31.700	54.5	9.1
ODP1233	31.772	54.6	9.6
ODP1233	31.844	54.7	9.8
ODP1233	31.916	54.8	9.8
ODP1233	31.988	54.9	9.7
ODP1233	32.078	55.1	9.9
ODP1233	32.179	55.2	9.9
ODP1233	32.280	55.3	10.2
ODP1233	32.382	55.4	10.1
ODP1233	32.483	55.6	9.6
ODP1233	32.584	55.7	9.1
ODP1233	32.660	55.8	10.2
ODP1233	32.685	55.8	10.0
ODP1233	32.786	55.9	10.4

ODP1233	32.887	56.0	9.7
ODP1233	32.988	56.2	10.0
ODP1233	33.089	56.3	10.1
ODP1233	33.216	56.4	9.6
ODP1233	33.266	56.5	10.4
ODP1233	33.368	56.6	10.4
ODP1233	33.469	56.7	10.0
ODP1233	33.570	56.8	9.7
ODP1233	33.671	57.0	9.7
ODP1233	33.772	57.1	9.5
ODP1233	33.873	57.2	10.0
ODP1233	33.974	57.3	9.4
ODP1233	34.075	57.4	9.5
ODP1233	34.168	57.6	9.1
ODP1233	34.269	57.7	9.0
ODP1233	34.370	57.8	8.6
ODP1233	34.472	57.9	8.9
ODP1233	34.573	58.0	9.0
ODP1233	34.674	58.2	8.4
ODP1233	34.876	58.4	9.4
ODP1233	34.977	58.5	8.7
ODP1233	35.078	58.6	9.2
ODP1233	35.180	58.8	9.6
ODP1233	35.264	58.9	8.8
ODP1233	35.365	59.0	9.9
ODP1233	35.459	59.1	9.9
ODP1233	35.546	59.2	9.8
ODP1233	35.633	59.3	10.0
ODP1233	35.720	59.5	9.6
ODP1233	35.808	59.6	9.4
ODP1233	35.895	59.7	9.7
ODP1233	35.982	59.8	9.9
ODP1233	36.069	59.9	10.0
ODP1233	36.156	60.1	9.2
ODP1233	36.243	60.2	9.7
ODP1233	36.853	61.0	10.6
ODP1233	36.940	61.1	11.7
ODP1233	37.027	61.3	11.3
ODP1233	37.114	61.4	10.9
ODP1233	37.201	61.5	9.9
ODP1233	37.288	61.6	8.9
ODP1233	37.325	61.7	10.0
ODP1233	37.412	61.8	9.8
ODP1233	37.499	61.9	9.2

ODP1233	37.586	62.0	9.0
ODP1233	37.673	62.1	8.8
ODP1233	37.760	62.3	9.6
ODP1233	37.847	62.4	9.4
ODP1233	37.935	62.5	10.1
ODP1233	38.003	62.6	9.3
ODP1233	38.072	62.7	10.4
ODP1233	38.140	62.9	10.3
ODP1233	38.209	63.0	11.0
ODP1233	38.277	63.1	10.9
ODP1233	38.294	63.1	10.8
ODP1233	38.363	63.3	9.6
ODP1233	38.431	63.4	10.4
ODP1233	38.500	63.5	10.7
ODP1233	38.574	63.6	10.7
ODP1233	38.643	63.7	10.5
ODP1233	38.711	63.9	10.2
ODP1233	38.780	64.0	11.0
ODP1233	38.848	64.1	11.2
ODP1233	38.917	64.2	11.7
ODP1233	38.986	64.3	9.6
ODP1233	39.054	64.5	10.7
ODP1233	39.123	64.6	10.5
ODP1233	39.191	64.7	9.9
ODP1233	39.261	64.8	9.6
ODP1233	39.341	64.9	10.0
ODP1233	39.501	65.2	8.8
ODP1233	39.581	65.3	8.9
ODP1233	39.821	65.7	8.3
ODP1233	40.062	66.0	7.9
ODP1233	40.576	66.8	8.0
ODP1233	40.983	67.4	8.6
ODP1233	41.063	67.5	8.6
ODP1233	41.223	67.8	8.3
ODP1233	41.402	68.2	9.4
ODP1233	41.612	68.9	9.1
ODP1233	41.654	69.0	9.0
ODP1233	41.943	69.8	10.3
ODP1233	41.985	69.9	9.4
ODP1233	42.026	70.1	8.9
ODP1233	42.233	70.7	10.0
ODP1233	42.468	71.3	8.8
ODP1233	42.481	71.4	8.9
ODP1233	42.719	72.1	8.4

ODP1233	42.733	72.1	9.2
ODP1233	42.857	72.5	9.2
ODP1233	42.982	72.8	9.7
ODP1233	43.230	73.5	10.9
ODP1233	43.534	74.3	10.0
ODP1233	43.685	74.6	9.9
ODP1233	43.972	75.3	8.3
ODP1233	44.128	75.7	7.6
ODP1233	44.280	76.0	8.0
ODP1233	44.466	76.4	7.7
ODP1233	44.674	76.7	7.9
ODP1233	44.883	77.1	8.6
ODP1233	45.069	77.4	9.8
ODP1233	45.277	77.8	10.0
ODP1233	45.486	78.1	10.0
ODP1233	45.701	78.5	10.6
ODP1233	45.886	78.8	11.3
ODP1233	46.095	79.2	11.1
ODP1233	46.292	79.5	9.8
ODP1233	46.501	79.9	9.7
ODP1233	46.710	80.2	8.9
ODP1233	47.098	80.9	9.7
ODP1233	47.307	81.3	10.2
ODP1233	47.725	82.0	10.4
ODP1233	48.123	82.7	9.2
ODP1233	48.516	83.4	8.5
ODP1233	48.898	84.1	9.0
ODP1233	49.269	84.8	9.1
ODP1233	49.662	85.5	8.8
ODP1233	50.410	86.9	9.3

Table S7. **$U^{K'}_{37}$ SST dataset of core GeoB7139-2.** The data were published in a previous study[3]. Note that the age model was recalibrated (see Table S12).

Age (kyr cal BP)	Depth (cm)	UK'37 SST (°C)
0.460	3	17.4
1.310	8	17.4
2.161	13	17.7
2.710	18	17.9
3.259	23	18.1
3.808	28	18.2
4.357	33	18.4
4.906	38	18.4

5.455	43	18.6
6.004	48	18.8
6.554	53	18.9
7.103	58	18.8
7.652	63	18.9
8.201	68	19.1
8.750	73	19.0
9.299	78	18.9
9.848	83	18.9
10.398	88	18.9
10.726	93	18.9
11.055	98	18.9
11.383	103	18.9
11.712	108	18.6
12.041	113	18.4
12.665	123	17.6
12.994	128	18.0
13.322	133	17.8
13.651	138	17.7
13.980	143	17.8
14.308	148	17.2
14.637	153	17.6
14.925	158	17.1
15.214	163	16.7
15.502	168	16.5
15.791	173	16.2
16.079	178	15.2
16.367	183	15.5
16.656	188	15.5
16.944	193	16.1
17.002	194	15.6
17.290	199	15.4
17.579	204	15.8
17.867	209	15.7
18.155	214	15.7
18.444	219	15.7
18.732	224	15.2
19.021	229	15.4
19.308	234	14.9
19.596	239	15.5
19.883	244	15.7
20.171	249	15.8
20.257	250	15.7
20.545	255	14.7

20.833	260	14.7
21.120	265	14.8
21.408	270	14.5
21.696	275	14.5
21.983	280	14.2
22.271	285	14.1
22.559	290	14.2
22.961	297	15.5
23.249	302	15.4
23.537	307	14.3
24.112	317	14.8
24.400	322	15.4
24.687	327	15.6
24.975	332	15.8
25.191	337	15.5
25.407	342	15.4
25.623	347	15.0
25.839	352	14.6
26.055	357	14.7
26.271	362	14.5
26.487	367	14.1
26.703	372	14.1
26.919	377	14.3
27.135	382	14.3
27.351	387	14.3
27.567	392	14.1
27.783	397	13.5
27.999	402	14.7
28.215	407	14.7
28.431	412	15.3
28.798	421	14.8
29.090	426	14.8
29.382	431	14.9
29.674	436	15.1
30.199	445	15.6
30.491	450	15.0
30.783	455	14.8
31.075	460	14.9
31.280	463	15.3
31.572	468	15.3
31.864	473	15.1
32.156	478	15.0
33.382	499	15.5
33.674	504	15.1

33.966	509	15.2
34.258	514	15.1
34.870	519	15.2
35.482	524	15.5
36.094	529	15.5
36.706	534	15.2
37.318	539	15.1
37.930	544	15.3
38.542	549	14.8
39.154	554	15.3
39.766	559	15.3
40.377	564	15.4
40.989	569	14.8
41.601	574	15.1
42.213	579	15.1
42.825	584	15.7
43.743	592	15.2
44.355	597	15.0
44.967	602	13.8
45.579	607	15.0
46.191	612	14.8
46.803	617	15.0
47.415	622	15.1
48.027	627	15.2
48.639	632	15.2
49.251	637	15.6
49.863	642	15.5
50.475	647	15.3

Table S8. **Humidity index dataset of core GeoB7139-2.** Note that the age model was recalibrated (see Table S12).

Age (cal ka BP)	Depth (cm)	Humidity index
0.290	2	0.31
0.630	4	0.32
0.970	6	0.31
1.310	8	0.25
1.650	10	0.25
2.380	15	0.32
2.490	16	0.29
2.710	18	0.27
2.929	20	0.30
3.149	22	0.31

3.369	24	0.27
3.588	26	0.30
3.808	28	0.21
4.028	30	0.32
4.247	32	0.25
4.467	34	0.19
4.687	36	0.19
4.906	38	0.28
5.126	40	0.22
5.345	42	0.21
5.565	44	0.16
5.785	46	0.15
6.004	48	0.16
6.224	50	0.23
6.444	52	0.11
6.663	54	0.21
6.883	56	0.18
7.103	58	0.25
7.322	60	0.18
7.542	62	0.19
7.762	64	0.25
7.981	66	0.27
8.201	68	0.29
8.421	70	0.29
8.640	72	0.26
8.860	74	0.32
9.080	76	0.25
9.299	78	0.21
9.519	80	0.32
9.739	82	0.25
9.958	84	0.19
10.178	86	0.23
10.398	88	0.31
10.529	90	0.23
10.660	92	0.24
10.792	94	0.20
10.923	96	0.24
11.055	98	0.20
11.186	100	0.24
11.318	102	0.30
11.449	104	0.30
11.581	106	0.23
11.712	108	0.27
11.844	110	0.27

12.106	114	0.30
12.238	116	0.24
12.369	118	0.18
12.501	120	0.28
12.632	122	0.15
12.764	124	0.18
12.895	126	0.24
13.027	128	0.20
13.158	130	0.18
13.290	132	0.22
13.421	134	0.28
13.552	136	0.27
13.684	138	0.32
13.815	140	0.25
13.947	142	0.14
14.078	144	0.33
14.210	146	0.37
14.341	148	0.42
14.473	150	0.39
14.604	152	0.34
14.724	154	0.35
14.839	156	0.41
14.954	158	0.32
15.070	160	0.32
15.185	162	0.35
15.300	164	0.29
15.416	166	0.23
15.531	168	0.30
15.646	170	0.25
15.762	172	0.27
15.877	174	0.26
15.992	176	0.35
16.108	178	0.38
16.223	180	0.48
16.338	182	0.45
16.454	184	0.46
16.569	186	0.47
16.685	188	0.44
16.800	190	0.34
16.915	192	0.45
17.031	194	0.52
17.146	196	0.49
17.261	198	0.62
17.377	200	0.32

17.492	202	0.44
17.607	204	0.69
17.723	206	0.67
17.838	208	0.74
17.953	210	0.71
18.069	212	0.69
18.184	214	0.74
18.300	216	0.54
18.415	218	0.60
18.530	220	0.59
18.646	222	0.69
18.761	224	0.60
18.876	226	0.69
18.992	228	0.71
19.107	230	0.70
19.222	232	0.62
19.337	234	0.65
19.452	236	0.73
19.567	238	0.62
19.682	240	0.61
19.797	242	0.66
19.912	244	0.55
20.027	246	0.64
20.142	248	0.32
20.257	250	0.75
20.372	252	0.77
20.488	254	0.76
20.603	256	0.60
20.718	258	0.62
20.833	260	0.55
20.948	262	0.49
21.063	264	0.50
21.178	266	0.37
21.293	268	0.40
21.408	270	0.47
21.523	272	0.52
21.638	274	0.53
21.753	276	0.41
21.868	278	0.70
21.983	280	0.65
22.098	282	0.61
22.213	284	
22.329	286	0.55
22.444	288	0.51

22.559	290	0.73
22.674	292	0.71
22.789	294	0.62
22.904	296	0.35
23.019	298	0.37
23.134	300	0.59
23.249	302	0.47
23.364	304	0.49
23.479	306	0.66
23.594	308	0.60
23.709	310	0.58
23.824	312	0.42
23.939	314	0.50
24.054	316	0.24
24.170	318	0.60
24.285	320	0.61
24.400	322	0.56
24.515	324	0.56
24.630	326	0.65
24.745	328	0.65
24.860	330	0.67
24.975	332	0.64
25.061	334	0.54
25.148	336	0.51
25.234	338	0.61
25.321	340	0.45
25.407	342	0.69
25.493	344	0.70
25.580	346	0.68
25.666	348	0.61
25.753	350	0.68
25.839	352	0.54
25.925	354	0.64
26.012	356	0.69
26.098	358	0.82
26.185	360	0.61
26.271	362	0.73
26.357	364	0.57
26.444	366	0.57
26.530	368	0.43
26.617	370	0.60
26.703	372	0.47
26.789	374	0.48
26.876	376	0.43

26.962	378	0.34
27.048	380	0.45
27.135	382	0.47
27.221	384	0.44
27.308	386	0.35
27.394	388	0.29
27.480	390	0.43
27.567	392	0.31
27.653	394	0.31
27.740	396	0.29
27.826	398	0.29
27.912	400	0.37
27.999	402	0.38
28.085	404	0.27
28.172	406	0.25
28.258	408	0.39
28.344	410	0.44
28.431	412	0.06
28.517	414	0.21
28.604	416	0.29
28.690	418	0.28
28.776	420	0.29
28.886	422	0.32
29.002	424	0.32
29.119	426	0.30
29.236	428	0.25
29.353	430	0.20
29.470	432	0.11
29.586	434	0.36
29.703	436	0.34
29.820	438	0.33
29.937	440	0.26
30.054	442	0.13
30.170	444	0.19
30.287	446	0.24
30.404	448	0.20
30.521	450	0.28
30.637	452	0.33
30.754	454	0.25
30.871	456	0.36
30.988	458	0.30
31.105	460	0.32
31.221	462	0.17
31.338	464	0.13

31.455	466	0.29
31.572	468	0.27
31.689	470	0.30
31.805	472	0.30
31.922	474	0.31
32.039	476	0.30
32.156	478	0.31
32.273	480	0.27
32.389	482	0.24
32.506	484	0.23
32.623	486	0.27
32.740	488	0.27
32.857	490	0.24
32.973	492	0.34
33.090	494	0.35
33.207	496	0.18
33.324	498	0.25
33.440	500	0.27
33.557	502	0.29
33.674	504	0.26
33.791	506	0.19
33.908	508	0.25
34.024	510	0.16
34.141	512	0.22
34.258	514	0.21
34.503	516	0.23
34.748	518	0.27
34.992	520	0.25
35.237	522	0.17
35.482	524	0.30
35.727	526	0.32
35.971	528	0.24
36.216	530	0.26
36.461	532	0.28
36.706	534	0.26
36.951	536	0.29
37.195	538	0.28
37.440	540	0.34
37.685	542	0.36
37.930	544	0.30
38.174	546	0.38
38.419	548	0.30
38.664	550	0.26
38.909	552	0.20

39.154	554	0.25
39.398	556	0.25
39.643	558	0.19
39.888	560	0.30
40.133	562	0.29
40.377	564	0.00
40.622	566	0.17
40.867	568	0.14
41.112	570	0.19
41.357	572	0.22
41.601	574	0.20
41.846	576	0.21
42.091	578	0.19
42.336	580	0.19
42.580	582	0.12
42.825	584	0.05
43.070	586	0.00
43.315	588	0.22
43.560	590	0.28
43.804	592	0.40
44.049	594	0.28
44.294	596	0.40
44.539	598	0.35
44.783	600	0.30
45.028	602	0.24
45.273	604	0.27
45.518	606	0.29
45.763	608	0.29
46.007	610	0.32
46.252	612	0.45
46.497	614	0.40
46.742	616	0.47
46.986	618	0.50
47.231	620	0.40
47.476	622	0.43
47.721	624	0.34
47.966	626	0.37
48.210	628	0.35
48.455	630	0.46
48.700	632	0.38
48.945	634	0.32
49.189	636	0.29
49.434	638	0.27
49.679	640	0.36

49.924	642	0.39
50.169	644	0.44

Table S9. **Fe/Ca dataset of core GeoB3375/15016[4].** Note that the age model was recalibrated (see Table S13).

Age (cal ka BP)	Depth (cm)	logFe/Ca
3.675	2	-0.435
7.400	4	-0.476
8.214	6	-0.518
9.028	8	-0.550
9.841	10	-0.521
10.655	12	-0.584
11.271	14	-0.616
11.688	16	-0.629
12.105	18	-0.625
12.522	20	-0.582
12.939	22	-0.557
13.356	24	-0.544
13.773	26	-0.505
14.190	28	-0.483
14.607	30	-0.460
15.024	32	-0.459
15.326	34	-0.378
15.513	36	-0.370
15.699	38	-0.343
15.886	40	-0.333
16.072	42	-0.323
16.259	44	-0.299
16.445	46	-0.271
16.632	48	-0.182
16.818	50	-0.166
17.004	52	-0.174
17.191	54	-0.172
17.377	56	-0.179
17.564	58	-0.158
17.750	60	-0.162
17.937	62	-0.169
18.123	64	-0.176
18.310	66	-0.180
18.496	68	-0.172
18.683	70	-0.144
18.869	72	-0.163

19.056	74	-0.159
19.242	76	-0.156
19.429	78	-0.153
19.640	80	-0.135
19.852	82	-0.128
20.063	84	-0.144
20.275	86	-0.135
20.487	88	-0.143
20.698	90	-0.140
20.910	92	-0.139
21.121	94	-0.114
21.333	96	-0.148
21.545	98	-0.159
21.756	100	-0.158
21.968	102	-0.158
22.179	104	-0.130
22.391	106	-0.134
22.603	108	-0.102
22.814	110	-0.092
23.026	112	-0.098
23.237	114	-0.109
23.449	116	-0.116
23.661	118	-0.114
23.933	120	-0.078
24.206	122	-0.142
24.478	124	-0.187
24.751	126	-0.179
25.024	128	-0.207
25.296	130	-0.213
25.569	132	-0.226
25.829	134	-0.221
26.077	136	-0.176
26.325	138	-0.161
26.573	140	-0.190
26.821	142	-0.159
27.070	144	-0.148
27.318	146	-0.178
27.566	148	-0.210
27.814	150	-0.214
28.062	152	-0.245
28.310	154	-0.247
28.558	156	-0.350
28.806	158	-0.425
29.054	160	-0.468

29.302	162	-0.478
29.887	164	-0.499
30.807	166	-0.491
31.727	168	-0.513
32.647	170	-0.544
33.567	172	-0.549
34.487	174	-0.571
35.407	176	-0.528
36.327	178	-0.530
37.247	180	-0.471
38.167	182	-0.376
38.723	184	-0.417
38.913	186	-0.322
39.103	188	-0.351
39.294	190	-0.456
39.674	194	-0.463
39.865	196	-0.439
40.055	198	-0.470
40.245	200	-0.420
40.436	202	-0.510
40.822	204	-0.433
41.403	206	-0.454
41.984	208	-0.392
42.565	210	-0.369
43.146	212	-0.376
43.699	214	-0.370
44.222	216	-0.359
44.746	218	-0.343
45.269	220	-0.401
45.793	222	-0.388
46.316	224	-0.371
46.840	226	-0.380
47.363	228	-0.399
47.887	230	-0.373
48.410	232	-0.338
48.934	234	-0.366
49.457	236	-0.323
49.981	238	-0.323
50.504	240	-0.355

Table S10. **GeoB7196-1 age model.** It is based on radiocarbon dates of the planktonic foraminifera *Globigerina bulloides* calibrated with the Marine20 calibration curve[5].

Depth (m)	Reservoir age (ka)	^{14}C age (ka)	±Error (ka)	SHCal20 calibrated age (cal ka BP)	±Error (ka)
0				-0.050	
35	0.400	0.870	0.020	0.334	0.137
66	0.400	1.400	0.025	0.788	0.123
156	0.400	2.095	0.020	1.492	0.142
215	0.400	2.655	0.020	2.178	0.144
245	0.400	3.020	0.060	2.610	0.204
305	0.400	3.420	0.020	3.110	0.162
335	0.400	3.710	0.020	3.471	0.141
365	0.400	4.135	0.020	4.002	0.162
395	0.400	4.410	0.020	4.370	0.163
425	0.400	4.640	0.020	4.670	0.153
525	0.400	5.215	0.025	5.402	0.147
625	0.400	6.050	0.030	6.280	0.150

Table S11. **ODP Site 1233 age model[2]**. It has been updated by recalibrating the radiocarbon dates with the Marine20 calibration curve[5]. An age of -0.050 cal ka BP has been attributed to the core top. The dating at 67.8 mcd is based on the Laschamp geomagnetic excursion[2].

Composite depth (mcd)	Reservoir age (ka)	^{14}C age (ka)	±Error (ka)	SHCal20 calibrated age (cal ka BP)	±Error (ka)
0				-0.050	
2	0.400	1.220	0.030	0.618	0.111
2.88	0.400	1.970	0.050	1.377	0.155
4.47	0.400	3.460	0.040	3.164	0.177
5.68	0.400	4.390	0.040	4.337	0.186
7.15	0.400	4.830	0.060	4.934	0.239
7.88	0.400	6.170	0.080	6.414	0.217
10.55	0.400	8.300	0.060	8.655	0.234
12.94	0.400	9.340	0.080	9.948	0.265
14.21	0.400	9.870	0.050	10.733	0.249
17.01	0.400	10.800	0.070	12.069	0.310
20.22	0.950	12.280	0.070	13.038	0.202
21.39	0.980	13.180	0.060	14.044	0.256
25.1	1.320	14.420	0.110	15.433	0.351
27.97	0.810	15.750	0.070	17.747	0.308
29.81	0.810	17.070	0.110	19.195	0.325
31.47	0.810	17.810	0.090	20.116	0.305
33.51	0.810	18.520	0.130	20.963	0.406
36.56	0.810	19.740	0.140	22.445	0.367
39.5	0.810	21.080	0.150	23.861	0.408
41.17	0.810	21.440	0.240	24.322	0.579
43.72	0.810	22.930	0.230	25.884	0.502

45.53	0.810	23.880	0.170	26.816	0.403
47.25	0.810	25.280	0.240	28.203	0.521
49.11	0.810	26.100	0.290	29.114	0.653
50.72	0.810	26.520	0.300	29.454	0.597
55.02	0.810	29.030	0.390	32.036	0.938
59.03	0.810	32.190	0.580	35.416	1.163
67.8				41.250	

Table S12. **GeoB7139-2 age model**[3]. It has been updated by recalibrating the radiocarbon dates with the Marine20 calibration curve[5]. An age of -0.050 cal ka BP has been attributed to the core top.

Depth (m)	Reservoir age (ka)	^{14}C age (ka)	±Error (ka)	SHCal20 calibrated age (cal ka BP)	±Error (ka)
0				-0.050	0.000
13	0.400	2.645	0.040	2.161	0.164
88	0.400	9.650	0.080	10.398	0.241
153	0.400	12.960	0.080	14.637	0.361
229	0.400	16.490	0.080	19.021	0.275
332	0.400	21.630	0.120	24.975	0.375
421	0.400	25.470	0.220	28.798	0.511
514	0.400	30.600	0.420	34.258	0.882
559	0.400	36.160	1.210	39.766	2.185

Table S13. **GeoB3375/15016 age model**[4]. It has been updated by recalibrating the radiocarbon dates with the Marine20 calibration curve[5]. An age of -0.050 cal ka BP has been attributed to the core top. The ages at 4 and 410 m were obtained by tuning the $\delta^{18}O$ of benthic foraminifera on the global stack[4].

Depth (m)	Reservoir age (ka)	^{14}C age (ka)	±Error (ka)	SHCal20 calibrated age (cal ka BP)	±Error (ka)
0				-0.050	0.000
4				7.400	0.000
13	0.400	10.160	0.100	11.062	0.328
33	0.400	13.340	0.080	15.233	0.289
78	0.400	16.840	0.110	19.429	0.362
118	0.400	20.460	0.330	23.661	0.771
133	0.400	22.350	0.210	25.705	0.470
163	0.400	26.090	0.330	29.427	0.652
183	0.400	34.860	1.140	38.628	2.321
203	0.400	36.870	1.220	40.531	1.702
213	0.400	40.610	2.900	43.437	5.315
410				95.000	0.000

References

1. Chikaraishi, Y. & Naraoka, H. Compound-specific δD-$\delta^{13}C$ analyses of *n*-alkanes extracted from terrestrial and aquatic plants. *Phytochemistry* **63**, 361–71 (2003).

2. Kaiser, J. & Lamy, F. Links between Patagonian Ice Sheet fluctuations and Antarctic dust variability during the last glacial period (MIS 4-2). *Quaternary Science Reviews* **29**, 1464–1471 (2010).

3. Kaiser, J., Schefuß, E., Lamy, F., Mohtadi, M. & Hebbeln, D. Glacial to Holocene changes in sea surface temperature and coastal vegetation in north central Chile: high versus low latitude forcing. *Quaternary Science Reviews* **27**, 2064–2075 (2008).

4. Lamy, F., Chiang, J. C. H., Martínez-Méndez, G., Thierens, M., Arz, H. W., Bosmans, J., Hebbeln, D., Lambert, F., Lembke-Jene, L. & Stuut, J.-B. W. Precession modulation of the South Pacific westerly wind belt over the past million years. *Proceedings of the National Academy of Sciences of the United States of America* **116**, 23455–23460 (2019).

5. Heaton, T., Köhler, P., Butzin, M., Bard, E., Reimer, R., Austin, W., Ramsey, C. B., Grootes, P. M., Hughen, K. A., Kromer, B., Reimer, P. J., Adkins, J., Burke, A., Cook, M. S., Olsen, J. & Skinner, L. Marine20—The Marine Radiocarbon Age Calibration Curve (0–55,000 cal BP). *Radiocarbon* **62**, 779–820 (2020).
